# Randomised trial of cord clamping at very preterm birth: outcomes at 2 years

**DOI:** 10.1136/archdischild-2019-316912

**Published:** 2020-04-15

**Authors:** Lindsay Armstrong-Buisseret, Katie Powers, Jon Dorling, Lucy Bradshaw, Samantha Johnson, Eleanor Mitchell, Lelia Duley

**Affiliations:** 1 Nottingham Clinical Trials Unit, University of Nottingham, Nottingham, UK; 2 Division of Neonatal-Perinatal Medicine, Dalhousie University, Halifax, Canada; 3 Department of Health Sciences, University of Leicester, Leicester, UK

**Keywords:** neonatal care with cord intact, outcomes at 2 years corrected age

## Abstract

**Objective:**

To report outcomes at 2 years corrected age for children of women recruited to a trial comparing alternative policies for timing of cord clamping and immediate neonatal care at very preterm birth.

**Design:**

Parallel group randomised (1:1) trial.

**Setting:**

Eight UK tertiary maternity units.

**Participants:**

Two hundred and seventy-six babies born to 261 women expected to have a live birth before 32^+0^ weeks’ gestation.

**Interventions:**

Deferred cord clamping (≥2 min) and immediate neonatal care with cord intact or immediate (≤20 s) clamping and immediate neonatal care after clamping.

**Main outcome measure:**

Composite of death or adverse neurodevelopmental outcome at 2 years corrected age.

**Results:**

Six babies born after 35^+6^ weeks were excluded. At 2 years corrected age, outcome data were not available for a further 52 children, leaving 218 for analysis (115 deferred clamping, 103 immediate clamping). Overall, 24/115 (21%) children allocated deferred clamping died or had an adverse neurodevelopmental outcome compared with 35/103 (34%) allocated immediate clamping; risk ratio (RR) 0.61 (95% CI 0.39 to 0.96); risk difference (RD) −13% (95% CI −25% to −1%). Multiple imputation for missing data gave an RR 0.69 (95% CI 0.44 to 1.09) and RD −9% (95% CI −21% to 2%).

**Conclusions:**

Deferred clamping and immediate neonatal care with cord intact may reduce the risk of death or adverse neurodevelopmental outcome at 2 years of age for children born very premature. Confirmation in larger studies is needed to determine the real benefits and harms.

**Trial registration number:**

ISRCTN21456601.

**What is already known on this topic?**

- A short delay in umbilical code clamping may reduce the risk of death before hospital discharge at very preterm birth.
- Immediate neonatal care can be provided with the cord intact, allowing cord clamping to be deferred for longer in babies requiring resuscitation at birth.
- Previous trials have been small, and few have reported outcomes beyond discharge from hospital.

**What this study adds?**

- Neurodevelopmental assessment at 2 years corrected age is feasible using a range of strategies, including routine clinical data.
- Deferred clamping with immediate neonatal care, if needed, may reduce the risk of death or adverse neurodevelopmental outcome at 2 years of age.
- Large high-quality trials and meta-analysis of individual participant data from these trials are needed to confirm the true benefits and harms.

## Introduction

Although just 1.1% of live births in the UK are very preterm (before 32 weeks’ gestation), these infants account for 43% of neonatal deaths.^1^ Those who survive are at increased risk for a range of neurodevelopmental sequelae including cerebral palsy, neurosensory and cognitive impairment, and attention, social and emotional problems.^2 3^ Such difficulties can have a major impact on a child’s health, well-being and academic attainment and may persist into adulthood.^4 5^ Interventions that could provide even a modest improvement in long-term outcomes would be of substantial benefit to these children and their families.

Systematic reviews have suggested that deferring clamping of the umbilical cord at preterm birth may reduce the risk of intraventricular haemorrhage (IVH)^6^ and death before hospital discharge.^6 7^ However, the trials largely excluded infants requiring immediate resuscitation at birth, and for very preterm births, most trials deferred clamping for 60 s or less. Also, data on long-term safety are sparse. The Cord Pilot Trial compared alternative policies for cord clamping and immediate neonatal care for very preterm births and results to discharge have been reported.^8^ This paper presents follow-up of the children after discharge and the results of neurodevelopmental assessments at 2 years corrected age.

## Methods

**Figure 1:**
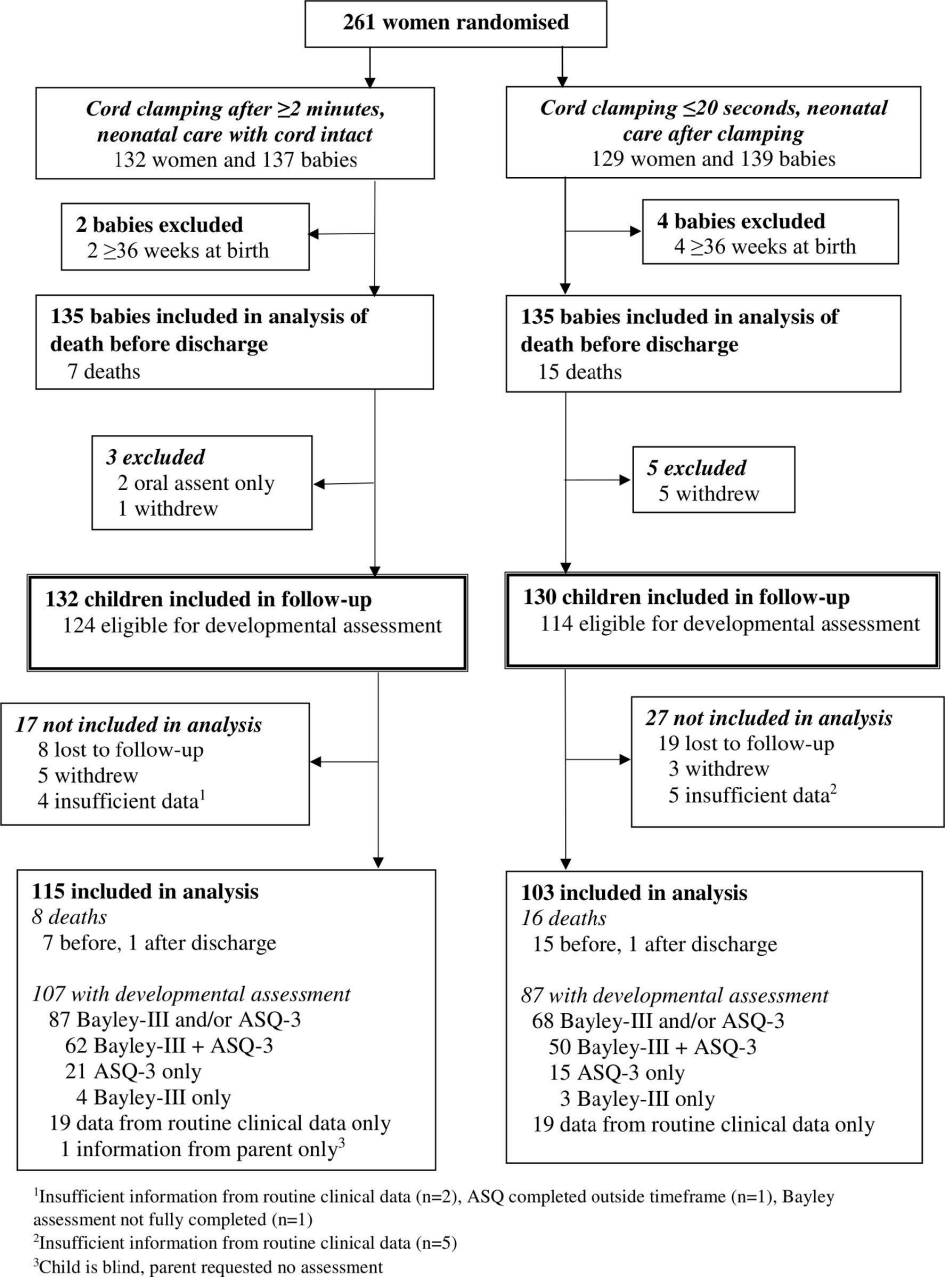
Flow for follow-up of children at 2 years (corrected age). ASQ-3, Ages and Stages Questionnaire-3.

The Cord Pilot Trial was conducted at eight UK hospitals, and the protocol is published.^9 10^ Women expected to have a live birth before 32^+0^ weeks’ gestation (very preterm) were randomised 1:1 either to deferred cord clamping after at least 2 min and, if needed, immediate neonatal care with cord intact or to immediate clamping within 20 s and neonatal care after clamping. To ensure women could be recruited when birth was imminent, we included a two-stage consent pathway, with oral assent for recruitment followed by written consent after the birth, alongside the usual one stage pathway (described in detail elsewhere^8 9^). Between March 2013 and February 2015, 261 women were randomised with six excluded as birth was after 35^+6^ weeks.^8^ Of the remaining 255 women, 17 had a twin pregnancy, for two of which one fetus died in utero before randomisation, leaving 270 children for the analysis of outcomes at hospital discharge.^8^ Of these, 22 babies died before discharge (including three stillbirths), and a further two died after discharge, giving a total of 24 deaths. Eight children were excluded from follow-up either because the mother had already withdrawn consent (n=6) or she gave oral assent for recruitment,^8^ and subsequent consent for follow-up was not available (n=2). Therefore, 238 children were eligible for assessment (figure 1).

### Parent and parent representative involvement

Parent and parent representative involvement has been reported.^8^ For the follow-up, parent representatives contributed to study design and materials, including the process for contacting families.

### Neurodevelopmental assessment of children

Children were assessed using the parent-completed Ages and Stages Questionnaire-3 (ASQ-3)^11^ and the Bayley Scales of Infant and Toddler Development, Third Edition (Bayley-III).^12^ If neither assessment was conducted, routine clinical data for 2-year outcomes were obtained if available.

Shortly before children reached 2 years corrected age, the ASQ-3 was posted to the mother along with an invitation for a Bayley-III assessment. If a completed ASQ-3 was not returned, the family was contacted with up to two reminder letters and a phone call. If no ASQ-3 was returned, parents were asked to complete the appropriate version (24, 27 or 30 months) during the home visit prior to the Bayley-III assessment or during a hospital visit. If >1 ASQ-3 was returned, the one completed closest to the date the child was 2 years corrected age was used for analysis.

The ASQ-3 includes 30 questions covering five domains: communication, fine motor skills, gross motor skills, problem solving and personal-social skills. The response to each question is ‘not yet’, ‘sometimes’ or ‘yes’ (scored 0, 5 and 10, respectively). For each domain, scores are summed to produce a total. The domain score is not calculated if ≥3 responses are missing. If one or two responses are missing, the domain score is calculated using the mean of the completed items to impute the missing items. Four further questions to assess hearing, vision and gross motor function were added to the ASQ-3 questions.^13^

The Bayley-III comprises three scales to assess cognitive, language and motor development.^12^ Children were assessed during a home visit by a trained researcher (KP) blind to intervention allocation. Three assessments were video recorded and scored by an independent observer (SJ) to assess inter-rater reliability.

### Outcome measures

The main outcome was a composite of death or adverse neurodevelopmental outcome at 2 years corrected age. Secondary outcomes included the individual components of the composite.

### Classification of adverse neurodevelopmental outcome

Neurodevelopmental outcomes were classified using the Bayley-III scores if available.^14 15^ If the child did not have a Bayley-III assessment, or individual scales could not be completed, ASQ-3 data plus our additional questions on hearing, vision and gross motor function were used. To account for underestimation of developmental delay using the Bayley-III, moderate or severe impairment was based on scores >1 SD below the normative mean (scores <85).^14 15^ For each ASQ-3 domain, developmental delay was based on a total score >2 SDs below the mean using published norms.^11^

Children were classified as having an adverse neurodevelopmental outcome if they met the criteria for a moderate/severe impairment in any one of five functions: motor, cognitive, speech/language, hearing or vision (see online supplementary appendix 1).^16^ For motor, this was defined as a Bayley-III gross motor scale score <7. If this scale had not been completed, assessment was based on our additional gross motor function questions from the ASQ-3, that is, if the child was unable to walk without assistance or sit without support. For cognitive and speech/language functions, this was defined as having a composite score <85 on the relevant Bayley-III scale. If these scales had not been completed, assessment was based on a score below the relevant domain cut-off on the ASQ-3 (problem solving for cognition and communication for language). Hearing and vision were assessed, respectively, as moderate/severely impaired if the child required hearing aids or was deaf, or had difficulty seeing with glasses, could only see light or was blind.

10.1136/archdischild-2019-316912.supp1 Supplementary data

### Blinded review of neurodevelopmental outcome

A blinded review of outcome data was undertaken if: clinical follow-up data only were available, the Bayley-III was not fully completed, the ASQ-3 was not completed within the correct timeframe or the additional questions included on the ASQ-3 about hearing and vision were not completed. For each child, data were assessed independently by two reviewers blind to the allocated group. Discrepancies were resolved by discussion or if needed by a third independent reviewer.

### Statistical analyses

As this was planned as a pilot trial, there was no formal power calculation.^9 10^ All analyses are based on the groups as randomly allocated (intention to treat) and were carried out using Stata SE 15.1. The main outcome (death or adverse neurodevelopmental outcome) is summarised by allocated group and presented with a risk ratio (RR), risk difference (RD) and 95% CIs. Each component of the main outcome is also summarised by group.

Multiple imputation using chained equations^17^ was used as a sensitivity analysis to include children with missing outcome data in order to explore their potential impact on the estimate of RR and RD. Variables included in the imputation model were maternal age, gestation at birth, mode of delivery, sex, receiving breast milk at discharge, necrotising enterocolitis, grade of IVH, bronchopulmonary dysplasia, treatment for retinopathy of prematurity and country specific decile of Index Multiple Deprivation.^18^ Thirty datasets were imputed, and estimates were combined using Rubin’s rules.^17^ This sensitivity analysis assumed that missing outcomes were missing at random,^19^ that is, conditional on the observed variables the distribution of the observed and missing outcomes are assumed to be the same.

## Results

Of the 238 children eligible for assessment, 27 were lost to follow-up, consent was withdrawn for a further 8 and for 9, there were insufficient data for analysis (figure 1). Therefore, 194 children were assessed at 2 years corrected age. As there were 24 deaths, 218 children were included in the analysis of the main outcome (115 allocated deferred clamping (≥2 min); 103 allocated immediate clamping (≤20 s)) (figure 1).

### Baseline characteristics and outcomes to discharge

Baseline characteristics and outcomes at discharge were similar for children included in the analysis of outcomes to discharge (n=270) and those included in the analysis of outcomes at 2 years (n=218) (table 1). For children included in analysis of outcomes at 2 years, baseline characteristics remained balanced between the allocated groups, with a median gestation at birth of 29 weeks (table 1).

**Table 1:** For children included in analysis of outcomes to discharge and outcome at 2 years, characteristics at entry and outcomes to discharge

	Included in analysis of outcomes to discharge		Included in analysis of outcomes at 2 years	
	Clamp ≥2 min	Clamp ≤20 s	Clamp ≥2 min	Clamp ≤20 s
	n=135 (%)	n=134 (%)*	n=115 (%)	n=102 (%)*
Women’s age (years) mean (SD)	30.5 (6.3)	29.4 (6.7)	30.1 (5.8)	29.8 (6.6)
Gestation at birth (weeks)				
Median (25th, 75th centile)	29 (27.1, 30.7)	29.1 (27.6, 30.4)	29 (27.1, 30.7)	29 (27.0, 30.3)
Two-stage consent pathway	36 (27)	37 (28)	30 (26)	31 (30)
Twin pregnancy	12 (9)	20 (15)	10 (9)	14 (14)
Caesarean delivery	86 (64)	73 (54)	74 (64)	56 (55)
Baby sex				
Male	71 (53)	72 (54)	58 (50)	60 (59)

For live births	n=134	n=132	n=114	n=100
Any IVH (grade 1–4)	43 (32)	47 (36)	36 (32)	35 (35)
Severe IVH (grade 3 or 4)	6 (4)	7 (5)	4 (4)	5 (5)
Periventricular leucomalacia	7 (5)	8 (6)	6 (5)	5 (5)
Blood transfusion	63 (47)	68 (52)	53 (46)	58 (58)
Jaundice requiring treatment	123 (92)	120 (91)	105 (92)	89 (89)
Chronic lung disease†	40 (31)	39 (33)	31 (28)	31 (35)
Ventilation	100 (75)	103 (78)	84 (74)	80 (80)
Necrotising enterocolitis	8 (6)	5 (4)	7 (6)	4 (4)
Sepsis‡	72 (54)	80 (61)	59 (52)	63 (63)
Treatment for patent ductus arteriosis	20 (15)	20 (15)	15 (13)	19 (19)
Duration of hospital stay (nights)§				
Median (25th, 75th centile)	57 (39, 85)	57 (37, 75)	57 (38, 80)	60 (40, 83)
Receiving mother’s breast milk at discharge§¶	71 (55)	68 (57)	61 (56)	55 (63)

*One woman withdrew (outcome data reported only for death before discharge).

†For babies surviving to 36 weeks postmenstrual age. n=129 clamping ≥2 min, n=120 clamping ≤20 s for all children and n=109, n=88 respectively for children included in analysis at 2 years.

‡Clinical sepsis defined as positive culture and ≥5 days antibiotics or negative culture and ≥5 days antibiotics.

§For babies alive at discharge n=128 clamping ≥2 min, n=120 clamping ≤20 s for all children and n=108, n=88 respectively for children included in analysis at 2 years.

¶Receiving breast milk at discharge not known for three babies in the clamp cord ≥2 min group (two included in analysis at 2 years) and one baby in clamp cord ≤20 s group (not included in analysis at 2 years).

IVH, intraventricular haemorrhage.

### Neurodevelopmental assessment

Data were available for 194/238 (82%) children alive at 2 years corrected age (table 2). Bayley-III data were available for 119/238 (50%) children, with a higher percentage allocated deferred clamping (66/124, 53%) than immediate clamping (53/114, 46%; table 2) being assessed. Corrected age at assessment was similar between groups. For 24 children with Bayley-III data, one or more scales were incomplete (13 deferred clamping, 11 immediate clamping); for 18, this was the motor scale. Inter-rater reliability of Bayley-III assessments was excellent with 97% agreement for the cognitive scale, 97% for language and 100% for motor (data not shown).

**Table 2:** For children included in follow-up and eligible for neurodevelopmental assessment (at 2 years corrected age), information about the assessment

	Clamp ≥2 min+neonatal care with cord intact	Clamp ≤20 s +neonatal care after clamping
	n=124 (%)	n=114 (%)
Any neurodevelopment data	107 (86)	87 (76)
Bayley-III and/or ASQ-3	87 (70)	68 (60)
Bayley-III+ASQ-3	62	50
Bayley-III only	4	3
ASQ-3 only	21	15
Clinical data only	20 (16)*	19 (17)
Age (corrected) at Bayley-III (months)		
<24	2	7
≥24 to <27	53	37
≥27 to <30	9	9
≥30	2	-
ASQ-3 version (months)		
24	78	60
27	3	3
30	2	2
No neurodevelopmental data	17 (14)	27 (24)

*Information from parent only for one child.

ASQ-3, Ages and Stages Questionnaire-3.

ASQ-3 data were available for 148/238 (62%) children, again with a higher response for those allocated deferred clamping (83/124, 67%) than immediate clamping (65/114, 57%) (table 2). For most children (138), the 24-month ASQ-3 was completed. The main reason for having neither Bayley-III nor ASQ-3 data was no response to the invitation to participate (72 children).

Routine clinical data were obtained for 39/238 (16%) children with neither ASQ-3 nor Bayley-III data (20 deferred clamping, 19 immediate clamping; table 2).

For children allocated deferred clamping, there was some evidence that those lost to follow-up had poorer outcomes at discharge than those who were assessed (table 3). In this group, a greater percentage of children with no neurodevelopmental data had IVH, blood transfusion or chronic lung disease compared with those with data. This was not observed for children allocated immediate clamping where there was some evidence of the opposite trend (table 3).

**Table 3:** For children alive at 2 years (corrected age) outcomes at discharge according to availability of developmental outcome data and allocated group

	Clamp ≥2 min+neonatal care with cord intact		Clamp ≤20 s +neonatal care after clamping	
	Neurodevelopmental data not available n=20 (%)	Neurodevelopmental data available n=107 (%)	Neurodevelopmental data not available n=32 (%)	Neurodevelopmental data available n=87 (%)
Any IVH (grade 1–4)	7 (35)	30 (28)	12 (38)	30 (34)
Severe IVH (grade 3 or 4)	2 (10)	–	2 (6)	3 (3)
Periventricular leucomalacia	1 (5)	5 (5)	3 (9)	3 (3)
Blood transfusion	10 (50)	46 (43)	10 (31)	45 (52)
Jaundice requiring treatment	18 (90)	98 (92)	31 (97)	80 (92)
Chronic lung disease	9 (45)	29 (27)	8 (25)	31 (36)
Ventilation	16 (80)	77 (72)	23 (72)	68 (78)
Necrotising enterocolitis	1 (5)	6 (6)	1 (3)	2 (2)
Sepsis*	13 (65)	55 (51)	17 (53)	54 (62)
Treatment for patent ductus arteriosis	5 (25)	15 (14)	1 (3)	16 (18)
Duration of hospital stay (nights)				
Median (25th, 75th centile)	64 (48, 105.5)	57 (38, 80)	46 (36, 63.5)	60 (40, 83)
Receiving mother’s breast milk at discharge†	10 (50)	61 (57)	13 (41)	54 (62)

*Clinical sepsis defined as positive culture and ≥5 days antibiotics or negative culture and ≥5 days antibiotics.

†Receiving breast milk at discharge not known for two babies in the clamp cord ≥2 min group with main outcome data available and one baby in both groups with no main outcome data available.

IVH, intraventricular haemorrhage.

### Death and adverse neurodevelopmental outcomes

Of children allocated deferred clamping, 24/115 (21%) died or had an adverse neurodevelopmental outcome compared with 35/103 (34%) allocated immediate clamping (table 4); RR 0.61 (95% CI 0.39 to 0.96); RD −13% (95% CI −25% to −1%). Using multiple imputation to account for loss to follow-up gave RR 0.69 (95% CI 0.44 to 1.09) and RD −9% (95% CI −21% to 2%).

**Table 4:** Death or adverse neurodevelopmental outcome at age 2 years (corrected)

	Clamp ≥2 min+neonatal care with cord intact n=115 (%)	Clamp ≤20 s+neonatal care after clamping n=103 (%)	Risk difference (95% CI)	Risk ratio (95% CI)
Death or adverse neurodevelopmental outcome	24 (21)	35 (34)	−13% (−25% to −1%)	0.61 (0.39 to 0.96)
Death	8 (7)	16 (16)	−9% (−17% to 0%)	0.45 (0.20 to 1.00)
Adverse neurodevelopmental outcome	16 (14)	19 (18)	−5% (−14% to 5%)	0.75 (0.41 to 1.39)
For children alive at 2 years, type of adverse neurodevelopment outcome*				
Motor	2 (2)	5 (6)		
Cognitive	11 (10)	6 (7)		
Hearing	2 (2)	2 (2)		
Speech/language	9 (8)	14 (16)		
Vision	2 (2)	2 (2)		

*For: clamp ≥2 min+neonatal care with cord intact n=107; clamp ≤20 s+neonatal care after clamping n=87.

In the deferred clamping group, 8/115 (7%) children died compared with 16/103 (16%) in the immediate clamping group (table 4). Of these, 3 were stillborn (1 deferred clamping, 2 immediate clamping), 19 died before discharge from hospital (6 deferred clamping, 13 immediate clamping) and 2 died after discharge (one in each group). Of children alive at 2 years, 16/107 (15%) allocated deferred clamping had an adverse neurodevelopmental outcome compared with 19/87 (22%) allocated immediate clamping.

Of children with a neurodevelopmental assessment, the most common type of adverse outcomes were in speech/language and cognitive impairment (table 4). Summary statistics for the 24-month ASQ-3 and Bayley-III are available in online supplementary appendix 2.

10.1136/archdischild-2019-316912.supp2 Supplementary data

## Discussion

Follow-up of children in the Cord Pilot Trial at 2 years corrected age suggests that deferring cord clamping for at least 2 min and providing immediate neonatal care, if needed, with cord intact may reduce the risk of death or adverse neurodevelopmental outcome compared with immediate clamping (≤20 s) and neonatal care after clamping. However, these results were sensitive to imputation to account for missing data. More children allocated deferred clamping had neurodevelopmental assessment data available; however, these children appeared to have fared slightly better at hospital discharge than those with missing follow-up data. In the immediate clamping group, there was evidence of the opposite trend when outcomes at discharge were compared according to availability of neurodevelopmental assessment data. Since the trial was not powered to demonstrate clinically important differences in outcome, confirmation is required in large high-quality randomised trials.

Strengths of our trial were that clamping was deferred for longer than in other trials at very preterm birth and that immediate neonatal care (including stabilisation and resuscitation), if needed, was provided with the cord intact.^20^ Providing neonatal care with the cord intact allowed high-risk babies needing immediate resuscitation at birth to be randomised, a group largely excluded from previous trials.^21^

A composite of death and adverse neurodevelopment was chosen as the main outcome, a decision supported by our parent representatives as being relevant to parents. Despite the problems of composite outcomes, this is considered acceptable for perinatal and neonatal trials provided it is likely that the direction of effect is the same for all components of the composite^22^ as is the case for alternative policies for cord clamping. In our trial, the overall reduction in death or adverse neurodevelopment in the deferred clamping group was primarily due to the reduction in death before discharge. There was no clear evidence of a difference between groups in adverse neurodevelopmental outcome or death after discharge.

Achieving high response rates for long-term follow-up is necessary to ensure sample representation; however, this is challenging. In order to achieve our 82% response rate, this necessitated employing multiple approaches and combining results obtained from different assessment tools, including diagnostic tests (Bayley-III), parent questionnaires (ASQ-3) and data obtained from routine clinical assessments. As screening tests may have poor diagnostic accuracy compared with gold standard tests^23^ and routine clinical assessments have poor sensitivity for evaluating cognitive outcomes in this population,^24^ this approach represents a limitation of our study. Future trials should attempt to attain long-term follow-up rates in excess of 90% and use a single standardised test to assess neurodevelopmental outcomes. Lower follow-up for children allocated immediate clamping may have been due to the women feeling they were not part of the trial as they received usual care or being disappointed they did not receive the ‘intervention’ of deferred clamping.^25^

Using routine data for developmental assessment in perinatal trials merits further evaluation as it is less intrusive for parents and less costly in terms of resources. In the UK, the National Institute for Health and Care Excellence has recently introduced guidance that high-risk children, such as those born before 30 weeks’ gestation, are eligible for enhanced developmental surveillance. This includes a developmental assessment at 2 years corrected age.^26^ Provided they are of sufficient quality, these data could be used to determine neurodevelopmental outcomes for children in future trials.

The most recent systematic review, which includes the Cord Pilot Trial, concluded that delayed clamping reduces hospital mortality compared with immediate clamping, with no clear effect on serious neonatal morbidity.^7^ This review emphasises the importance of follow-up for children recruited to both existing and future trials. The latter should be large enough to provide adequate power to detect clinically important differences in outcomes and will also need to achieve high follow-up rates to enable reliable comparisons of neurodevelopmental outcomes. Since providing robust evidence is likely to require multiple trials, a systematic review and individual participant data meta-analysis of cord management at preterm birth is underway.^27^

## Conclusions

Follow-up of children in the Cord Pilot Trial suggests deferred clamping with immediate neonatal care, if needed, beside the mother may reduce the risk of death or adverse neurodevelopmental outcome at 2 years corrected age compared with immediate clamping and neonatal care after clamping. Large high-quality trials are needed to confirm the true benefits and harms.
