# Supplementary material for: Randomised trial of cord clamping at very preterm birth: outcomes at 2 years
Source: Arch Dis Child Fetal Neonatal Ed. 2020 Apr 15;105(3):292–8. doi: 10.1136/archdischild-2019-316912 (PMC7363783; doi:10.1136/archdischild-2019-316912)
Supplement: Supplementary data [file fetalneonatal-2019-316912supp001.pdf]

Appendix 1: Criteria for moderate/severe impairment based on Bayley-III scores and responses to additional questions included on the ASQ-3

**Function**	**Criteria for moderate/severe impairment**
Motor	• Bayley-III gross motor scaled score < 7<br>• If Bayley-III not completed, based on additional questions 9 (*Is your child able to walk on his/her own without support?*) and 10 (*Is your child able to sit on the floor on his/her own, without any support?*) included on ASQ-3 if any of the following responses apply:<br>o *Can only walk if helped by an adult or a walking aid (Q9)*<br>o *Unable to walk, even with help (Q9)*<br>o *Can only sit with support or help from an adult (Q10)*<br>o *Unable to sit (Q10)*
Cognitive	• Bayley-III cognitive scale composite score < 85<br>• If Bayley-III not completed, score below cutoff on problem solving domain of ASQ-3
Speech/Language	• Bayley-III language scale composite < 85<br>• If Bayley-III not completed, score below cutoff on communication domain of ASQ-3
Hearing	• Additional question 7 (*Does your child have any problems with his/her hearing?*) included on ASQ-3 if any of the following responses apply:<br>o *Has a cochlear implant or hearing aid, but hears well with it*<br>o *Has difficulty hearing, even with a cochlear implant*<br>o *My child is deaf*
Vision	• Additional question 11 (*Does your child have any problems with his/her vision?*) included on ASQ-3 if any of the following responses apply:<br>o *Has difficulty seeing, even when wearing glasses*<br>o *Is blind in one eye but has good vision in the other*<br>o *Is able to see light only or is blind*
