# Supplementary material for: Randomised trial of cord clamping at very preterm birth: outcomes at 2 years
Source: Arch Dis Child Fetal Neonatal Ed. 2020 Apr 15;105(3):292–8. doi: 10.1136/archdischild-2019-316912 (PMC7363783; doi:10.1136/archdischild-2019-316912)
Supplement: Supplementary data [file fetalneonatal-2019-316912supp002.pdf]

Appendix 2: For children with available data, summary of ASQ-3 24 and Bayley-III at 2 years (corrected age)

		Clamp ≥2 minutes + neonatal care with cord intact	Clamp ≤20 seconds + neonatal care after clamping
**ASQ-3 24:**	median [25th, 75th centile]	n = 78	n = 60
	Communication[1]:	55 [40, 60]	50 [32.5, 60]
	Gross motor:	50 [40, 60]	50 [40, 60]
	Fine motor:	50 [45, 55]	50 [40, 55]
	Problem solving:	40 [35, 50]	40 [30, 50]
	Personal-social:	45 [35, 55]	45 [40, 55]
**Bayley-III:**	mean [sd]		
	Cognitive composite:	101.7 [17.1]	100.1 [15.2]
	n	66	53
	Language composite[2]:	103.8 [16.7]	96.2 [17.0]
	n	61	51
	Motor composite[3]:	100.6 [12.7]	97.6 [11.8]
	n	57	44

ASQ scores range from 0 to 60 with higher scores indicating greater ability. Median [25th, 75th centile] presented as distribution of the scores was left skewed.

Bayley composite scores standardised to have mean 100 and SD 15.

[1]Three questions missed (out of six) for communication domain on ASQ24 for one child, so score not calculated.

[2]Reasons language subtest not completed was due to English not being first language for 6 children and due to deafness for 1 child (clamp ≥2 minutes)

[3]17 children did not complete either fine motor or gross motor subtests due to fatigue. One child did not completed fine motor subtest due to being not cooperative (clamp ≥2 minutes)
